# A Typology of Moderator Practices and Community Guidelines in Online Mental Health Communities to Establish a Framework for Digital Safety: Systematized Review

**DOI:** 10.2196/99367

**Published:** 2026-10-21

**Authors:** Ashleigh Charles, Stefan Rennick-Egglestone, Karen L. Fortuna, Neil Coulson, Stine Bjerrum Moeller, Matthew Geeleher, Julian Harrison, Mark Holden, Mike Slade, Fiona Ng

**Affiliations:** 1 School of Health Sciences, Institute of Mental Health, University of Nottingham, Innovation Park Triumph Road, Nottingham, NG7 2TU, United Kingdom, 44 0115 823 1294; 2 NIHR Nottingham Biomedical Research Centre, Institute of Mental Health, Nottingham, United Kingdom; 3 Patient Innovation Lab, Department of Community and Family Medicine, Geisel School of Medicine at Dartmouth, USA, Lebanon, NH, United States; 4 School of Medicine, University of Nottingham, Nottingham, United Kingdom; 5 Institute of Psychology, University of Southern Denmark, Odense, Denmark; 6 Mental Health Services in the Region of Southern Denmark,, Department of Multidisciplinary Trauma Treatment, Middelfart, Denmark; 7 EPOCH Lived Experience Advisory Panel, Nottingham, United Kingdom; 8 Health and Community Participation Division, Faculty of Nursing and Health Sciences, Nord University, Postbox 474, Namsos, 7801, Norway

**Keywords:** online mental health communities, digital peer support, moderation, mental health, mental health forum

## Abstract

**Background:**

The rise of online mental health communities has reshaped the ways individuals seek and receive mental health support. Although online mental health communities (OMHCs) offer a valuable space for peer support, including through experience-sharing, they also present challenges, including moderation practices to cultivate supportive interactions and secure member safety. No comprehensive framework of moderation practices has been established. Developing such an understanding is important, as moderators play a central role in facilitating a sense of community and determining whether OMHCs function as safe and supportive spaces.

**Objective:**

This study aimed to develop typologies of moderator practices used to support safety and facilitate a sense of community within OMHCs for people with mental health problems, and the content of OMHC community guidelines.

**Methods:**

A systematized review of empirical research studies focusing on moderation practices and community guidelines in OMHCs was conducted using MEDLINE, Web of Science, Association for Computing Machinery Digital Library, supplemented with a purposive search to identify gray literature on community guidelines for OMHC moderation. Inclusion criteria: online, text-based component, asynchronous or synchronous moderation, mental health–related focus, and human moderation. Exclusion criteria: crisis-focused, audience-based, nonmoderated or bot-moderated, profession not peer support, non-English, and academic review or thesis. The search period was 2014 to 2024. A bespoke data extraction sheet was piloted, then populated, capturing document, OMHC, member, and moderator characteristics. Thematic analysis was used to synthesize the findings relating to each objective.

**Results:**

The systematized search identified 11,347 studies and other documents, from which 80 studies were included, comprising 48 empirical papers and 32 gray literature documents. These related to 52 different OMHCs. Four categories of moderator practices were identified: (1) premoderation and postmoderation processes, (2) reactive moderation processes, (3) building the community, and (4) monitoring members. Premoderation and postmoderation processes were used within 41 (79%) of the 52 OMHCs. Three categories of community guideline content were identified: (1) content of post, (2) member conduct, and (3) moderation. The category, content of post, was the most widely used component of community guidelines, used in 35 (67%) of the 52 OMHCs.

**Conclusions:**

The two typologies provide a foundational framework that can be adapted and applied to community guidelines and moderation strategies across a range of contexts. When operationalized, they could provide benefit to people with mental health problems by using them to guide the development of moderator training, digital tools, and governance policies that align with the specific characteristics and objectives of individual OMHCs. Effective translation and application of these typologies require careful, nuanced consideration of each community’s goals, membership, and cultural context to ensure their relevance, effectiveness, and impact.

## Introduction

The rise of online mental health communities (OMHCs) has transformed how individuals seek and receive mental health support. OMHCs are digital, often moderated, platforms in which individuals share experiences, gain support, and exchange information regarding mental health challenges. These digital spaces, hosted on dedicated websites and public and private social media platforms, provide accessible, asynchronous, and often anonymous environments where people can share experiences, seek advice, access peer support, and find emotional connection outside of traditional clinical settings [1]. Notably, online support groups for people with chronic conditions are increasingly used and show benefits for social well-being, positive behaviors and adjustment (eg, illness acceptance) [2].

Digital peer support interventions such as OMHCs show promising evidence for clinical effectiveness. When combined with evidence-based practices, there are improved outcomes in relation to symptomatology, self-management skills, and service use [3]. Participation in OMHCs alone has also been shown to offer forms of benefit similar to in-person talking therapies, including increased feelings of social connectedness, enhanced self-efficacy, and a greater sense of belonging [4,5]. Some advantages of these communities include anonymity, privacy, immediacy, and ease of access, as well as the promotion of help-seeking behaviors [1,6]. OMHCs have become an important component in the mental health landscape [7]. Their scalability, wide reach, and low cost make them particularly well-suited for public health strategies focused on prevention and early intervention, offering accessible mental health at a population level.

While OMHCs offer a valuable space for peer support and sharing experiences, they also pose significant challenges that require active, ongoing moderation. Conversations in these spaces can involve intense emotional disclosures and sensitive topics, including trauma, self-harm, and suicide [8]. Without appropriate oversight, such discussions may expose vulnerable individuals to potentially harmful content, including triggering material, misinformation, or escalating interactions. At the same time, moderation plays a broader role in shaping the tone and dynamics of the community, helping to cultivate supportive interactions and sustain a sense of safety and connectedness among participants. To mitigate the risks and foster a sense of community, moderators, who may be volunteers or paid peers, trained moderators, or health professionals, are essential in the effective functioning of these communities. Responsibilities may include screening content, maintaining safety, ensuring community guidelines are upheld, and fostering a respectful and supportive culture [9]. Their actions not only influence and facilitate individual interactions but also shape the community’s overall well-being and its capacity to provide meaningful support. However, if moderators fail to act in the best interests of individuals or the community, such as not intervening in harmful situations or enforcing guidelines inconsistently, this could result in harm, including exclusion or the undermining of trust within the community.

Despite the important role moderators play in managing and sustaining OMHCs, research examining the specific practices they use remains limited. Existing studies have primarily used qualitative methods to understand moderators’ experiences, including how they create supportive spaces for disclosure, ensure privacy [10], facilitate peer-to-peer support [11], and manage sensitive content related to self-harm and suicide [12]. These empirical studies identify specific challenges in OMHCs not found in other types of health-related communities, including content that is triggering [13] and moderator burnout [14]. As such, there is still limited understanding of the day-to-day practices through which moderators maintain safety, shape community dynamics, and foster a sense of community and engagement. While individual strategies have been examined, no published comprehensive framework of moderation practices has been identified [15]. Understanding these practices is important, as moderators shape whether OMHCs function as safe and supportive spaces. Addressing this knowledge gap will require a comprehensive understanding of moderator practices and the guidelines they use. This will inform the components of a moderator role, improve support for moderators, and strengthen their role within the broader mental health ecosystem.

This review was conducted as part of the Evaluating Positive Changes in Psychosis (EPOCH) study, which is developing a new digital intervention, including an OMHC, to support posttraumatic growth in psychosis. In developing the EPOCH intervention, we needed to understand what practices moderators undertake to support safety and foster a sense of community and engagement within an OMHC for people with mental health problems. In addition, we were interested in identifying the content included within OMHC’s community guidelines to inform the development of community guidelines for the EPOCH intervention.

The aim of this systematized review was to characterize how OMHCs maintain safety and encourage a sense of community. To support the development of typologies of moderation practices and community guidelines, we adopted a systematized approach to identifying and analyzing relevant literature and platform materials, rather than conducting a full systematic review of evidence. The objectives were to develop typologies characterizing (1) moderator practices, and (2) the content of community guidelines and rules relating to member behavior and interactions (OMHC guidelines). The intention is that these typologies bridge the gap between ad hoc moderation and standardized, evidence-based training and governance.

## Methods

### Study Design

A systematized review of empirical research articles on moderation was conducted to characterize the most widely used moderating practices and the most common components of community guidelines in OMHCs. Unlike a full systematic review, a systematized review approach includes some, but not all, of the elements associated with systematic reviews [16]. However, we enhanced the depth of the systematized review findings by purposively sampling gray literature, including community guidelines for OMHCs. This combined approach was selected because moderators adhere to community guidelines that are not always published or reported in academic literature and continually evolve in practice, providing valuable insights into how OMHCs are managed.

### Eligibility Criteria

Studies were included about moderation practices in OMHCs with the following features: (1) accessed via the internet, (2) the user interface has a text-based component, (3) moderation is asynchronous such as a forum or synchronous such as a chat room, (4) the OMHC focus is on providing support around mental health, including diagnostic-specific communities (eg, for people diagnosed with an anxiety disorder), experiential-based communities (eg, for people who hear voices), and general mental health communities (eg, aimed at people without a specific diagnosis but seeking support), (5) the OMHC has at least one human moderator. Gray literature documents were included if they could be accessed without a login or account creation and contained a list, code of conduct, behavior guidelines, community rules, or details regarding the moderator role. Literature was excluded if the OMHC was primarily for crisis support, audience-based (eg, centered around a content creator), nonmoderated or bot-moderated, or provided one-to-one support from a health care professional and thus lacked a peer support component. Literature was also excluded if it was not written in English or if the document was a review or thesis. The search covered the period between January 2014 and December 2024, reflecting the rapidly evolving landscape of OMHCs since 2014 including the increasing use of mental health chatbots [17].

### Information Sources

Three data sources were used:

1. Three electronic bibliographic databases (MEDLINE, Web of Science, and ACM Digital Library) were searched from the specific date onwards to identify peer-reviewed publications about OMHCs. Each included paper was checked for relevant supplementary documents and/or materials.
2. Google was searched for “online mental health community” to identify the most popular OMHCs, and the top ten hits were reviewed.
3. A review of social media platforms with the highest numbers of users [18] was used to identify informal OMHCs on these platforms. For each OMHC platform, “mental health” was searched using the internal search, and the 5 OMHCs with the highest monthly active users were screened.

The range of databases (n=3) and the decision to use the top 10 (Google) and 5 (social media platforms) hits reflect the systematized rather than systematic review methodology, which is intended to identify the most widely used rather than all OMHC moderator approaches.

All OMHCs identified in the search were screened individually for inclusion, and, where available, community guidelines were extracted.

Searches were conducted in April 2024, and the identified literature was collated using Covidence (Veritas Health Innovation Ltd.) software. The search involved initial screening by two reviewers (BRI and MM) of titles and abstracts, followed by full-text screening, with a 10% concordance check, sufficient to achieve high concordance. Any discrepancies were discussed and resolved collaboratively with the researchers. The full-text screening was subsequently reviewed by AC, with further checks and concordance discussions carried out with FN to ensure consistency. The review did not include ongoing database searches over time.

### Data Abstraction

A bespoke data extraction sheet was created and then piloted on 10 documents (5 empirical and 5 gray literature) by FN and AC. Modifications to the data extraction sheet included the addition, refinement, and expansion of characteristic categories. For example, the inclusion of “formality of OMHC” and the differentiation between “intervention,” “formal” and “informal” communities. The finalized data extraction form, shown in Multimedia Appendix 1, was used to record for each document:

1. Document characteristics, comprising title, year of publication, and publication type.
2. OMHC characteristics, comprising geographic region of the OMHC, name of OMHC, the level of OMHC formality, type of access, community purpose, and platform type.
3. Member characteristics, comprising criteria for membership, member age range, number of OMHC members, and level of anonymity for members.
4. Moderator characteristics, comprising moderation level, moderator background, and moderator remuneration.

### Analysis

Data synthesis was conducted using document analysis on all included documents, ie, academic papers, gray literature, and online web pages, involving skimming, reading, and interpretation of the data. This process followed guidance from Bowen [19] who recommends an iterative process involving content and thematic analysis. A 5-step process was followed. First, 2 analysts (AC and FN) independently categorized data relevant to each objective. For all included documents, verbatim text segments relating to moderator practices and community guidelines were extracted. Second, initial codes were developed through line-by-line analysis of extracted text segments by AC and 10% of codes were checked by FN. Codes for each objective (moderator practice and community guidelines) were differentiated and analyzed separately. Initial codes for a moderator’s practice were descriptive in nature, describing specific actions that moderators undertook. For community member guidelines, initial codes descriptively summarized each guideline and rule within included documents. Third, initial descriptive codes of a similar nature were categorized into subthemes by AC and checked by FN to allow for broader patterns of meaning to emerge. For example, specific mentions of guideline violations or issuing warnings or reminders before taking further action were categorized under “giving behaviour warnings.” Fourth, subthemes of a similar nature were grouped together by AC and checked by FN. The subthemes were reviewed by checking them against the whole data set, informing the merging and integrating of categories. Once confirmed, the label names were reviewed to ensure comprehensibility, eg, “account use” was changed to “account responsibility and security.” This coding framework was then used to create each typology, with all analysts contributing to final choices about the definition and organization of superordinate and subordinate categories and the choices of exemplar text. Finally, a vote counting of the number of documents identifying each category was conducted to determine the strength of the theme of each category, conducted by AC, to indicate how commonly each category was used. This strength of theme analysis did not influence the content of the typology. The 2 primary analysts (AC and FN) came from different academic (psychology and sociology) and mental health professional backgrounds (nursing) and were responsible for all data extraction and analysis. All disagreements were resolved through discussion, comparison with other coded data, and consensus. The other 7 analysts came from different disciplinary backgrounds (nursing, health service research, health psychology, mental health research, technology design research, human computer interaction, public involvement, equality, diversity, and inclusion, and lived experience) and supported the interpretation of themes and resolution of any coding disagreements. The s7 analysts were not involved in the coding of data.

## Results

### Overview

The systematized search identified 11,347 studies and other documents, from which 80 [11,20-96] met the inclusion criteria. The flow diagram is shown in Figure 1.

**Figure 1.**
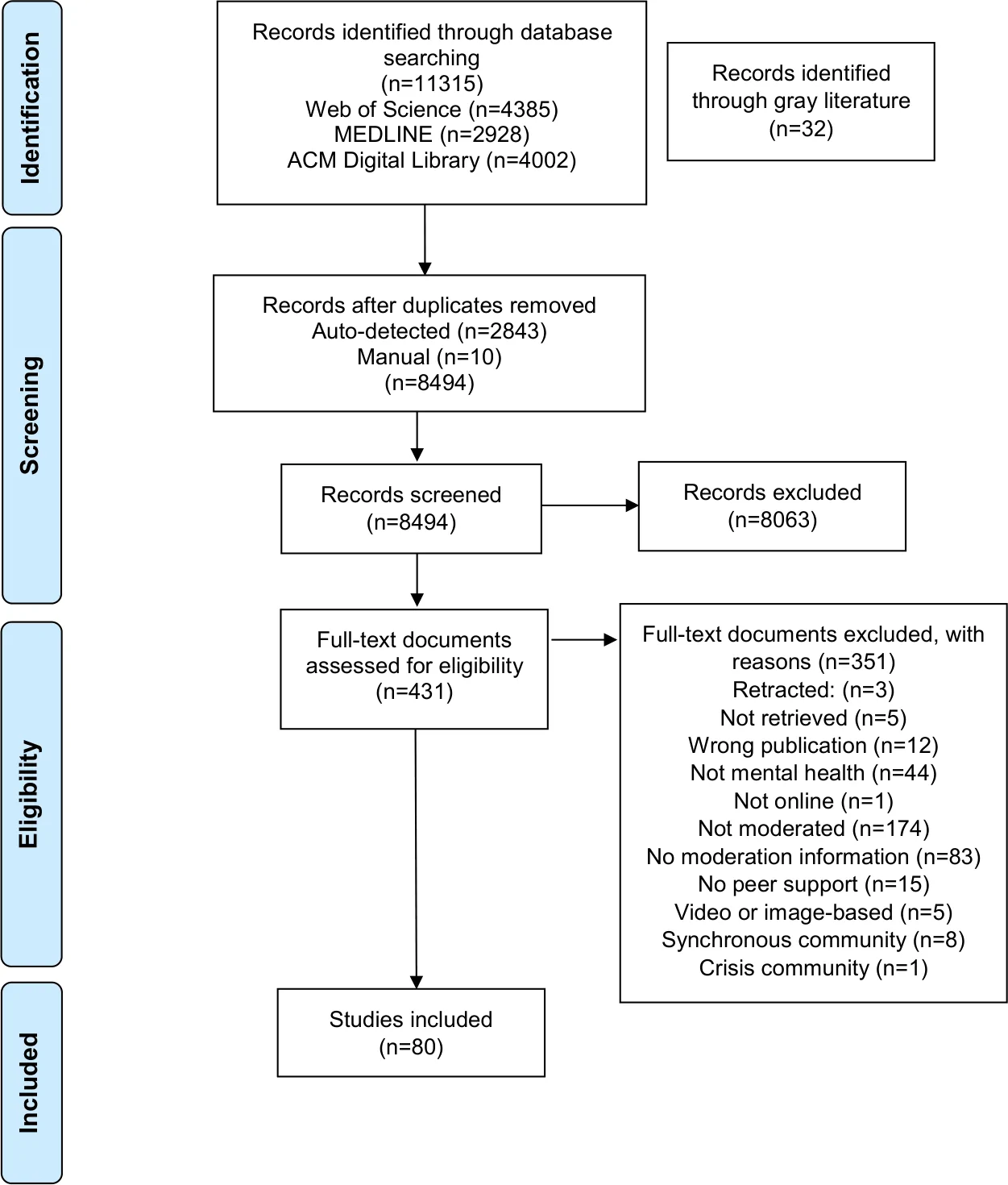
Flow diagram for included studies.

In total, 80 documents [11,20-96] were included, as detailed in Multimedia Appendix 2. Documents comprised academic papers (n=47) [11,20-63], webpages (n=29) [64-92], and others (n=4) [93-96], with only slightly more published 2021‐2025 than 2015‐2020. Among these, gray literature contributed 29 sets of community guidelines, compared to 9 sets from academic sources. The 80 sources identified discussed 52 distinct OMHCs, with 4 sources mentioning more than one OMHC. The 52 OMHCs included, their locations or the locations of their lead authors were distributed as follows: United Kingdom (n=12) [35,49,51,57,66,68,71,74,79,80,88], the United States (n=11) [38-40,42-44,65,69,76,90,97], Australia (n=10) [20,24,27,32,33,37,45,48,70,91], Canada (n=2) [23,41], the Netherlands (n=2) [26,60], Taiwan (n=1) [63], and the United Kingdom and North America (n=1) [75], with the remaining 13 OMHCs [61,62,73,77,78,81-87,89] having no geographic access restrictions. The OMHCs were hosted on various platforms, including private non–social media websites (n=30) [20,23,24,26,27,32,33,35,37,42-45,48,49,51,57,60,65,66,68-71,73-76,90,91], social media websites (n=20) [38-40,61,62,77-89,92,97], platforms that incorporate both private and social media components (n=1) [63], and one OMHC [41] that did not report its platform type.

Based on their descriptions, OMHCs were allocated to levels of formality: (1) formal (n=16) [48,49,51,57,65,66,68-71,73-76,90,91], which are professional or training spaces often linked to health care providers or organizations; (2) intervention (n=18) [20,23,24,26,27,32,33,35,37-45,97], targeted platforms delivering specific mental health interventions, usually guided by professionals or trained facilitators; and (3) informal OMHCs (n=18) [60-63,77-89,92], community-based or peer-led spaces where individuals share experiences and support each other, often without professional oversight. As shown in Multimedia Appendix 2, most guidelines were published for formal and informal OMHCs, rather than intervention OMHCs.

Four primary methods for accessing OMHCs were identified: (1) invitation-only groups (n=14) [20,23,24,26,27,32,33,35,37,39,40,45,60,75], such as intervention-related OMHCs for research purposes (n=12) [20,23,24,26,27,32,33,35,37,39,40,45], (2) publicly accessible content with sign-up required to post (n=18) [38,65,68,69,71,76,81-91,97], (3) closed groups (n=18) [42-44,48,49,51,61-63,66,70,73,74,77-80,92], for example, Facebook (Meta) closed groups which require application to join, (4) open community (n=1) [57] allowing free access to content and posts, and one OMHC access type was unspecified. The included OMHCs stated various community purposes related to mental health support, with some more general, focusing on overall mental health support (n=9) [49,51,62,66,70,85,87,88,92]. In contrast, others were more specific, targeting individuals with specific mental health problems (n=33) [20,23,24,26,27,32,33,35,37-45,57,60,61,63,65,76-80,82-84,86,89,97], and many combined both general and specialized support within their OMHC (n=10) [48,68,69,71,73-75,81,90,91].

### Objective 1: Typology of Moderator Practices

Different types of moderator characteristics were identified. Three approaches to moderation were identified: premoderation, occurring before the message is visible to other users; postmoderation, occurring after the message is visible to other users; and reactive moderation, occurring when complaints or concerns are raised about a specific post. Most OMHCs used multiple moderation approaches; the most common were postmoderation and reactive moderation (n=22) [20,27,32,35,37,39,41-43,45,48,51,57,60,65,70,71,73,75,85,88,90], followed by all 3 approaches (n=12) [24,26,33,38,44,62,63,74,83,86,89,97], premoderation and reactive moderation (n=2) [82,91], and premoderation and postmoderation (n=1) [68].

Moderators within the OMHCs had differing backgrounds, including health professionals (those with health professional registrations), trained moderators (those who have received some formal moderator training), peer moderators (who have lived experience of mental health problems or a specific diagnosis that is relevant to the OMHC), and volunteer moderators (who volunteer their time to moderate). Most OMHCs had peer moderators (n=13) [39,60,68,77-80,83,85-88,92], and many had a mixture of backgrounds, such as health professionals and peer moderators (n=5) [27,35,43,70,91] or health professionals and volunteer moderators (n=4) [69,71,81,90]. Remuneration practices varied: most were not reported (n=21) [20,23,24,26,27,32,33,35,37,39-43,48,60,61,65,69,76,81], the majority were volunteer roles (n=19) [38,51,62,63,68,77-80,82-89,92,97], some were paid roles (n=7) [44,45,49,57,66,74,75], and others had a mix of paid and volunteer roles (n=5) [70,71,73,90,91].

Moderator practices were synthesized into four categories, summarized in Table 1 and fully described in Supplementary Table S1 in Multimedia Appendix 2.

**Table 1.** Typology of OMHC^a^ moderator practices.

Moderator practice	Number of documents (N=52)	Definition of practice	Example practice text [reference and OMHC code in abstraction Table]
Premoderation and postmoderation processes (n=52)			
Screening content	52	Reviewing new member content, before or after posting, to ensure it aligns with OMHC guidelines and values, including ongoing monitoring to maintain a safe and supportive environment.	Almost all moderators use the preapproval tool to review the contents of the posts before they get published in the group [80, I18] Everything that you post or submit to the site is checked by staff members who moderate what you’ve written and make sure it doesn’t break any of the community boundaries [42, F6]
Clinical risk monitoring	23	Monitoring overall community activity to identify patterns or content that may indicate emerging risks or threats to the safety and well-being of the OMHC.	Moderators screen the system daily for evidence of clinical risk, including reports of evidence of deterioration in the mental state of the young person in the care of participants [3, V1] A study investigator logged in to the ISG daily to review new posts for suicidal thoughts and other potentially inappropriate content [29, V15]
Stigma monitoring	4	Challenging negative stereotypes or harmful beliefs expressed within the OMHC to foster a more respectful, inclusive, and supportive environment.	Gender-sensitised moderation strategies were also incorporated based on established literature, to boost engagement with the platform among young males [5, V3] Peer moderation serves to normalise experiences, counteract stigma and promote engagement [8, V5]
Boundary monitoring	17	Communicating the scope and limits of what the OMHC can offer, helping members understand what support is available and what falls outside the community’s role.	This site is not for crisis services. If you or your child has a psychiatric emergency, please contact one of the resources on our crisis page or call 911 [24, V10] We offer nonjudgemental peer support ONLY [76, I14]
Defining Moderator role boundaries	6	Outlining the scope and limits of the moderator’s responsibilities within the OMHC, including expected tasks and exclusions, the clear articulation of these boundaries to community members, and the establishment and communication of limits on personal and emotional involvement, to support the community while maintaining moderator well-being.	The Wall Guides also ensure that members do not form overly tight bonds with any singular member or with the Wall Guides themselves, which can lead to potentially unhealthy dependency, thus ensuring consistent utilization of peer support between community members [53, F14] The role of moderation does NOT include the provision of emotional support directly to members, including those who may be feeling suicidal or in crisis [46, F10]
Signposting to resources	8	Pointing members toward relevant resources and information, both within the OMHC (such as toolkits, guides, and dedicated spaces) and beyond it, to help them access the support, tools, and knowledge they need.	Their key role was to provide emotional support and to guide relatives to relevant parts of the toolkit or other resources, as appropriate [16, V8] What we tend to do is we’ll say: we’re really sorry we can’t publish this [.] but hey let’s have a talk about it, maybe here’s an NHS information page we can give you that you can read and come back if you have any questions’ [41, F6]
Reactive moderation processes (n=41)^b^			
Assessing flagged content	30	Responding to content or concerns flagged by members to assess potential risks or violations and take appropriate action to maintain community safety.	Content is not pre-approved, so please flag anything that you think needs our urgent attention [48, F11] If you have a concern about a post, select the red exclamation mark attached to the relevant post. All reports will be read; action may be taken as necessary [46, F10]
Content management decision	—^c^	Guidelines and procedures for evaluating flagged or reported content, determining whether it breaches community guidelines.	—
Editing and removing content	38	Editing content by taking actions such as blocking, hiding, muting, locking, moving posts, or removing specific words, and/or entirely removing or deleting member content that does not align with OMHC guidelines.	Moderation was intended to keep users of the forum safe, and posts were edited if the moderators believed they contravened these guidelines [58, F15] All User Content is moderated before going live [50, F13]
Restricting, suspending, and deleting member accounts	29	Taking account-level action in response to repeated or serious breaches of OMHC, including restricting accounts, such as locking posts, disabling comments, or limiting access to the forum at certain times; suspending accounts for a set period of time; or terminating or deleting accounts entirely.	Overstepping or violating any guidelines may result in behavioural points, warnings, account suspensions, or other restrictions as per Safety Factsheet or need [33, F1] We reserve the right to put forum members on moderation and/or remove their access to the private messaging system for any reason [43, F7]
Feedback process	—	Reviewing and responding to member feedback, appeals, or complaints about moderation decisions and the application of community guidelines.	—
Providing feedback	15	Explaining the reasons behind their decisions, such as why the content was edited.	Following suspensions or restrictions to a user’s account, the moderator will contact the young person at monthly intervals to ascertain whether the account is to be reactivated [8, V5]
Giving behavior warnings	10	Issuing warnings or reminders to members to address community guideline violations and encourage positive participation before taking further action.	We ask each user to live by the guidelines outlined in this thread. Each behavior mentioned will be assigned a behaviour point, once a certain number of points are accumulated within a specific period, consequences will be enacted [33, F1] Issue Members with verbal or written warnings and take such further action as we deem appropriate if such warnings are not heeded [52, F14]
Allowing appeals of decisions	6	Allowing members to appeal those decisions made by moderators if they disagree, and/or stating that feedback will not be provided.	Please contact the moderators if you disagree with a decision [67, I7]
Building the community (n=26)^d^			
Role-modeling	5	Demonstrating positive ways to engage with the OMHC, including respectful communication, help-seeking, and use of platform features, to guide and encourage member behavior.	Peer moderators, with lived experience of caring for a relative with psychosis, modelled system use and facilitated online interactions [2, V1]
Self-disclosing lived experience	4	Sharing aspects of their own lived experience to build trust, foster connection, and create a supportive environment for members.	Peer workers were peer moderators who facilitated social learning using Moderated Online Social Theory (MOST+) in desired ways (eg, self-disclosing) [32, V18]
Greeting new members	6	Welcoming new members to the community to foster a sense of belonging and encourage early engagement.	Moderators welcomed new users and encouraged existing users to interact with them within 24 hours of enrolment [21, V9] They roll out the virtual ‘welcome mat,’ enabling new members to feel at home and safe [54, F14]
Building content	—	Guidelines for developing, sharing, and engaging with content within the OMHC.	—
Creating content and facilitating interactions	23	Developing and distributing content within the OMHC to inform, guide, and support members, while also initiating and leading conversations that encourage active engagement and meaningful interaction.	The moderators are implicitly entrusted with establishing and maintaining an environment conducive to candid, respectful, and supportive dialog [81, I19] Participants and moderators can post comments, information, upload pictures and videos, ‘like’ different content, and interact with others in ways similar to other social networking platforms [6, V3] To facilitate the process of reflection on the content by participants, moderators could also contribute to the talking points [1, V1]
Responding to member content	16	Engaging directly with members by replying to their posts or comments to acknowledge contributions, offer support, or guide discussion.	The peers also frequently posted comments or provided encouragement in response to participants’ posts [26, V12] Trained “super peers” review posts and make sure no one is left without a response [51, F13]
Increasing OMHC engagement	—	Guidelines and strategies to encourage active participation and sustained involvement in the OMHC.	—
Encouraging use of the OMHC	13	Promoting ongoing participation and engagement in the community through supportive messaging and reinforcement of the OMHC’s value.	Sometimes if a member is too dependent on private messaging to us, Wall Guides, we will empathize and provide a reflection of what they are going through, but we have to push them to keep trying to use the community [53, F14] Young people with a lived experience of social anxiety provided online peer support on Entourage, to help users feel comfortable contributing to the platform and maintain engagement [5, V3]
Providing individual content suggestions	5	Recommending specific posts or activities tailored to individual members’ needs or interests to support their involvement and connection within the OMHC.	Moderators develop individualised formulations regarding users’ level of motivation to use Altitudes and how their specific needs can best be addressed to optimise system engagement [3, V1]
Using reminders, prompts, and notifications	8	Using scheduled prompts, reminders, or notifications to encourage regular member activity and engagement in the community.	To address engagement, we have embedded a “reminders” feature in the design of our platform to send students necessary reminders about online activities (eg, video-conferencing sessions) [28, V14] If engagement waned, moderators could utilise an SMS system built into the platform to facilitate reconnection of participants [3, V1]
Leading guided problem solving	6	Supporting members in exploring and addressing challenges by guiding them through problem-solving processes.	Entourage incorporates a “Talk it Out” feature, where users can post unique challenges relating to their symptom experience, which moderators then support through a problem-solving style discussion [5, V3] Peer workers guided the problem-solving discussions in Talk it Out and posted links to relevant therapeutic resources and tips [32, V18]
Prompting the use of learned skills	5	Encouraging members to apply strategies, tools, or techniques they have learned, and reinforcing helpful behaviors within the OMHC.	Prompted by moderators to practice their strengths or use skills they have learnt while engaging with the social network [8, V5]
Monitoring members (n=38)^e^			
Screening new members	6	Assessing and approving new member requests to ensure they align with the purpose and values of the OMHC before granting access.	One key role of moderators was to filter out non-CAPs who joined the site under false pretences, such as “pedo-hunters” or journalists that pretend to be paedophilic [78, I15] We are fully aware that several professional bodies have joined this group; however, before posting in the group, offering your professional support in therapy, or life coaching, etc Please message the head admin to verify your qualifications :) [63, I4]
Providing support	11	Offering various forms of support, including emotional and peer support, general encouragement, and informational support, to help members feel heard, valued, and supported within the OMHC.	The discussions were moderated by staff to address informational questions and to provide additional support as needed [27, V13]
Individual risk monitoring	16	Observing and assessing specific members who may be at risk of harm to themselves or others.	Clinical coaches conducted risk assessments in a one-to-one email or phone check-in when participants expressed significant distress on the forum, in group, or in weekly survey responses [4, V2] If any material you post raises concerns about your safety or the safety of others, we may try to contact you to make sure that you or others are safe. We may also need to pass your contact details on to authorities who can help protect your safety or the safety of others (for example, to the police or a mental health crisis service) [34, F3]
Signposting to mental health support	21	Guiding members toward appropriate external mental health services or crisis resources when their needs go beyond what the OMHC can provide.	Staff and moderators may ask you to seek professional, medical, or crisis support away from the community [45, F9] Neither our moderators nor other users are trained to provide support to people who are experiencing a crisis and would therefore urge you to contact the below: [organisation: number] [39, F5]
Contacting members and external agencies	22	Initiating contact with members (n=19), their emergency contacts (n=3), or external agencies such as safeguarding and social services (n=4), preventing violent extremism (PREVENT) (n=1), nonemergency teams, such as mental health team (n=4), emergency medical services (n=7), the police (n=7), and other relevant parties (eg, internet provider) (n=2) to support member safety.	I ask for email addresses (and the server logs IP addresses) so that if there’s abuse or serious danger, I can notify the person’s Internet service provider (or other authorities). [66, I6] If an immediate risk was identified, the REACT supporters called the police or social services, depending on the risk [16, V8]

a OMHC: online mental health community.

b Definition: reviewing flagged posts by platform systems or reported by community members as potentially harmful, inappropriate, or against community guidelines, and assessing whether they pose risks and deciding on the appropriate action.

c Not applicable.

d Cultivating a welcoming, supportive, and inclusive culture grounded in humanism, including modeling respectful communication, encouraging positive peer-to-peer support, recognizing valuable contributions, and facilitating a sense of belonging so members feel safe to share their experiences.

e Definition: focusing on individual members’ posting patterns and behaviors, especially if they show signs of escalating distress, repetitive concerning content, or boundary-crossing interactions, including balancing safeguarding (eg, stepping in with resources or interventions) with protecting the poster’s dignity and privacy.

### Objective 2: Typology of OMHC Guidelines

Some documents used the term “rule” to convey explicit injunctions or prohibitions, and others used “guidance” or “guidelines” to imply flexible recommendations. However, the content’s language was inconsistent, with some “rules” including recommendations, and some “guidelines” including requirements. There was a considerable variation in the depth, specificity, and scope of guidelines across different OMHCs. For example, although hate speech was prohibited across all included OMHCs, the definitions and descriptions varied substantially. Some communities provided highly detailed lists of protected attributes, including those related to disability, gender, sexual orientation, immigration status, religion, race, and political orientation. Others adopted broader, less prescriptive definitions, acknowledging that the dynamic nature of language makes it challenging to codify every form of harmful speech. The community guidelines were synthesized into 3 categories, summarized in Table 2 and fully described in Supplementary Table S2 in Multimedia Appendix 2.

**Table 2.** Typology of OMHC^a^ guidelines for members.

Community guideline	Number of documents (/52)	Definition of guideline and rule	Example guideline/rule text [reference and OMHC in Abstraction Table]
Content of post (n=35/52)^g^			
Content	—^c^	General rules governing the sharing of content.	—
External links	24	Rules on sharing links to external websites and services, including sharing personal blogs, social media accounts, or invitations to join other online groups (n=17), with some communities allowing this type of sharing, while others prohibit it.	This is not a venue to promote your blog or vlog [67, I7] We welcome you to post links to support groups, or links to online therapy, etc, or links to pages, etc [63, I4] In addition, for the safety of the members, the board does not permit posting of links to other providers [38, F4]
General image/video rules	21	General guidelines around what images and videos can be shared and posted, including rules around using audiovisual content (eg, GIFs, memes, and short videos) (n=9), screenshots (n=2), and personal photos and videos (eg, selfies or images of family or friends; n=7).	Any images posted must not contain moving images or GIFS [43, F7] Our photo gallery is for use by registered members. Members are free to post any photograph or image that they have the intellectual property rights to [44, F8]
Spam	18	Expectations around avoiding spam, including repetitive, irrelevant content, automated content or mass posting, including automated messages or unrelated promotional content.	Please don’t post spam [62, I3] Do not spam the same post or comment multiple times. If you post or comment the same content 3 or more times in a day, we will class this as spam and subsequently, this content will be removed [49, F12]
Copyrighted materials	7	Rules about the use of copyrighted text, images, media, and materials, including uploading content they don’t have the right to share, and how members should respect the intellectual property rights of the community platform itself.	You must not post media (images, text, video, audio, or other) on our Apps unless you have full ownership/rights in such media or are otherwise legally permitted to do so [50, F13] We respect the intellectual property rights of others, and we prohibit Members from uploading, posting or otherwise transmitting on the Site any materials that violate another party’s intellectual property rights [52, F14]
Trigger and content warnings	17	Rules around members using trigger or content warnings when sharing sensitive or potentially triggering content, as defined within the OMHCs guidelines, and/or avoiding posting graphic or explicit details.	Before posting a sensitive post that could a trigger to anyone.then please ensure you put warning (topic trigger) at the top of the post or top of the chat message, so people can scroll by. we just want to make sure everyone is safe on this amazing group xxxx [63, I4] Trigger Warnings. Please be mindful of common triggers. If your post or comment includes something that could be triggering, say so in your title or in the first line of your comment [67, I7]
Mental health-related content	—	Rules governing the sharing of mental health-related content within the OMHC.	—
Self-harm	20	Rules around self-harm-related posts, including what members are permitted to share about self-harm behaviors or methods, recognizing some OMHCs may allow supportive, non-graphic discussion, while others restrict or prohibit content such as forbidding posts that promote, normalize, or glorify self-harm/injury, or that contain details, instructions, dares, challenges, or encouragement.	NO methods for any type of self-harm, or validating self-destructive intent [70, I9] Self-harm- we won’t publish any posts that contain details of methods of self-harm or descriptions of self-harm injuries [34, F3]
Suicide	20	Rules around suicide-related posts, including what members are allowed to share about suicidal ideation, thoughts, plans, or methods, acknowledging some OMHCs permit limited, supportive discussion, while others prohibit content entirely, such as forbidding posts that promote, normalize, encourage, or glorify suicide, or share graphic details or instructions.	Stating that you experience suicidal thoughts or have been feeling suicidal is not banned here; however, this sub is not able or suitable to appropriately manage those who are actively suicidal, are intending to act on their thoughts and not receptive to support/advice [75, I13] Rule 17. Do not share content that depicts, promotes, normalizes, encourages or glorifies suicide or self-harm [49, F12]
Death	2	Rules around what members are allowed to post about death, dying, or grief, acknowledging some OMHCs may allow open, supportive sharing, while others set stricter limits on detail and tone; these rules often prohibit graphic descriptions or content that romanticizes death.	Sensitive topics such as death, suicide and self-harm should be flared with a Trigger Warning [73, I11]
Experiences of abuse	16	Rules around what members can post about experiences or threats of abuse, violence, or harm. These rules generally forbid reference to physical, emotional, sexual, or psychological abuse, domestic violence, or threats or acts of harm toward oneself or others.	Experiences of abuse or trauma - details of experiences of abuse and trauma are best discussed with counsellors, you can share with others your stories of overcoming difficult events, but we won’t be able to publish any specific details [42, F6]
Psychoactive substances	14	Rules on members sharing content about alcohol, drugs, medications, and other psychoactive substances, distinguishing between personal experiences and the promotion of medications (n=8) and between personal experiences and the promotion of unregulated or illicit substances (n=10). While some OMHCs allow harm-reduction discussions, most prohibit promotional, instructional, or advertising content related to substance use.	When discussing illegal drugs or alcohol, try to avoid specifics about quantities or illegal drug use [39, F5] No talk about specific drugs or how to obtain them. Please refrain from sharing specific drug names or dosages prescribed to you by your medical professional [74, I12] Rule 59. Do not post or share content that promotes or encourages use of drug consumption, manufacturing or selling [49, F12]
Food and eating	13	Rules around what members may share about dieting, eating behaviors, body weight, measurements, or body image, recognizing some OMHCs permit supportive conversation around healthy habits, while others limit or prohibit such topics, such as banning posts that promote or glorify disordered eating behaviors, sharing tips, coaching, or encouragement around restrictive eating.	Please don’t share specific methods or behaviours associated with disordered eating (such as fasting, purging or laxative abuse). This can be harmful and triggering for other users. Please don’t share specific information about body weight, body measurements or weight-loss methods [45, F9]
Gambling	2	Rules around what members can share about gambling, recognizing some OMHCs may be specific to gambling and thus allow discussion of recovery and support, while most prohibit any discussion or promotion of gambling, betting, gaming for money or valuables, including the sharing of strategies, links, or individual experiences.	Rule 58. Do not post or share content that promotes gambling [49, F12] We ask members not to talk about gambling or share links to gamble sites, unless they are sharing experiences around addiction or seeking support around gambling issue [48, F11]
Medical advice	19	Rules around the sharing of medical, diagnostic, or professional advice or information, including instructions, details, specific names, or recommendations about treatments, medications, or their side effects.	No medical assistance is to be provided here [74, I12] Please do not diagnose or prescribe medicine here - even if you are licensed! Do not cancel or change your treatment regimen based solely on advice from this subreddit [73, I11]
Misinformation	13	Rules that prohibit members from posting false, inaccurate, misleading, or unverified claims about mental health, treatments, or related topics, recognizing that different OMHCs define misinformation in varying ways, and from presenting opinions as facts, sharing conspiracy theories, or offering advice that is not evidence-based or could be unhelpful, distressing, or harmful to others.	No medical misinformation [59, F16] No misinformation. This includes: Stigma towards any particular diagnosis, harmful non fact-based advice in risky areas such as in the management of psychotic symptoms, harmful generalisations about groups of people or their perceived intentions including mental health professionals, and speculating around someone’s diagnosis/non-diagnosis unless they are specifically asking about that or post strongly indicates openness to that (though preferably to make clear you are not able to diagnose them even if qualified) [75, I13]
Polarizing topics	—	Rules governing the sharing of content that may be upsetting, triggering, or emotionally challenging for members.	—
Illegal activity	11	Rules around what members can share regarding illegal acts, acknowledging that some OMHCs allow general discussion for awareness or prevention; most prohibit content that encourages, describes, or facilitates illegal behavior.	7. No Illegal activity [59, F16] Please don’t use this site to facilitate any illegal activities [66, I6]
Politics	10	Rules around what members are permitted to share about political topics, with some OMHCs allowing respectful discussion, and others limiting or prohibiting any political content, including political figures, ideologies, events, governments, news discussions, individual opinions, or political advocacy.	No Overtly or Needless Political or Religious Posts. Any political or religious discussion without any direct link to a mental health issue will be deleted. The moderators reserve the right to lock the post if it goes controversial [73, I11] Politics. /Anxiety attempts to be as politically neutral as possible, and we ask that our users respect that. Threads about politically-based anxiety should focus on the anxiety [67, I7]
Religion, spirituality, and personal beliefs	8	Rules around what members can share about religion, spirituality, or personal beliefs, recognizing some OMHCs may allow open discussion, while others limit or prohibit to avoid divisiveness, such as any content discussing beliefs, practices, or institutions.	All moms’ groups ‘forbid sensitive topics,’ including politics and religion, because such discussions could be offensive to other group members with opposing positions [81, I19] Topics/Posts NOT Permitted Religion [61, I12]
Extremism	2	Rules around what members are permitted to post about terrorism or extremism, acknowledging some OMHCs may allow factual or educational discussion, most restrict or prohibit content promoting terrorist ideologies, extremist beliefs, or violence for ideological, political, or religious purposes.	Tellmi has a commitment to counter terrorism and will not allow posts or replies that may show allegiance or support to terrorist organisations or extremist groups [50, F13]
Minors	3	Rules around sharing images or videos of children or minors.	We ask that members do not post: Images of children which are identifiable- for example, in a school uniform [48, F11]
Sexual and graphic images	14	Rules prohibiting the sharing of sexual or suggestive images or videos (eg, real and stylised content featuring nudity, implied sex acts, or offers of sexual services (n=13) and graphic images or videos (eg, containing graphic and/or offensive content, including explicit violence, self-injury, cruelty, gore, or depictions of abuse; n=5).	Please do not upload images containing profanity or nudity, or imagery of a sensitive nature that could potentially trigger other users [46, F10] Please don’t post any images relating to self-injury, these could be upsetting for other users [34, F3]
Advertising	30	General rules on what members may post regarding advertising content, including personal or commercial products, businesses, or services (n=19), some OMHCs may allow certain types of advertising, while others prohibit all advertising content or all forms of self-promotion.	Any advertisements or affiliate links will be deleted [73, I11] No self-advertising or posting about products or services without approval from the Moderators [69, I8]
Jobs	1	Rules on posting about job openings or paid opportunities, with all included, prohibiting this.	No posts on job openings allowed. Anyone posting about jobs would be banned from the group [60, I1]
Private practice promotion	4	Rules regarding therapists or practitioners promoting their private practice (eg, links to services, offering sessions, or recruiting clients) vary across OMHCs, with most prohibiting this.	If you’re a professional mental health expert, you’re welcome to engage in peer support but don’t offer therapy on our platform [49, F12]
Events	3	Rules on sharing events, such as mental health–related events (eg, workshops, webinars), differ between OMHCs, with most prohibiting this.	It is ok to post: General meet up spots/locations in communal places [48, F11] We NEVER allow; Surveys Polls Petitions In person/meetup events [61, I2]
Funding Requests	10	Rules regarding requests for donations, funding, or financial support (including GoFundMe or similar platforms) differ with some OMHCs allowing such requests, while others do not.	This is not the place to request money or share your story for the purposes of any financial gain [74, I12] Fundraisers can be promoted but only on behalf of mental health charities [75, I13]
Research	15	Rules on members asking members to take part in surveys or studies for research purposes; some OMHCs may require members to contact the moderators for approval to conduct research (eg, evidence of obtaining ethical approval), while others have a strict rule against posting any research-related content.	Using the forum for research purposes is not permitted unless you are a member and have explicitly been granted permission through our research approval process [43, F7] No advertisements of mental health surveys or studies. Do not post a survey or study related to mental health in our subreddit [69, I8]
Member conduct (n=41)^d^			
Welcoming new members	5	Guidelines that encourage members to welcome new members in the community.	If you see a new member, please take a minute to say hi. They might feel scared, and a warm welcome might help them relax and partake. And they’ll be more likely to come back! [66, I6] At any time, the community will host established and new members, as well as people considering whether to join. Please keep an eye out for new members, acknowledge introductory posts, and welcome new members [46, F10]
Communication style	—	Guidelines that define how members engage with one another.	—
Respectful communication and conduct	39	Guidelines around how members are expected to engage with kindness, inclusivity, and respect, avoiding inappropriate conduct such as personal attacks, bullying, harassment, threats, derogatory remarks, promoting harmful behaviors, or posting while under the influence (n=35). This also includes members avoiding hostility, harsh criticism, “tough love” approaches, arguments, rumors, or gossip (n=7). While disagreements and debates may occur, they should remain constructive, with members valuing diverse perspectives (n=15) and focusing on respectful dialogue, problem-solving, and positive conflict resolution (n=4).	Be kind and courteous. We’re all in this together to create a welcoming environment. Let’s treat everyone with respect. Healthy debates are natural, but kindness is required [63, I4] You agree not to post any abusive, obscene, vulgar, slanderous, hateful, threatening, sexually-oriented or any other material that may violate any applicable laws [44, F8] It’s okay to disagree as long as we stay respectful [69, I8]
Giving and receiving support	26	Guidelines that emphasize the goal of the OMHC in providing a space where members share their feelings and experiences, listen to one another, and offer support to build understanding and connection.	We offer non-judgemental peer support ONLY [76, I14] This community thrives on peer support, so please give back when you can. One small comment or reaction from you could make a huge difference to someone’s day [48, F11]
Responding to posts	6	Guidelines for members reading and responding to others’ experiences or stories, including being respectful, sensitive, nonjudgmental engagement, avoiding assumptions or giving diagnoses, and recognizing that everyone’s experience is unique.	Do NOT post general “uplifting” or anti-suicide messages. Encouragement should never be offered until you’ve demonstrated understanding of the OP’s emotions and experience, to their satisfaction, not yours! [76, I14] Be sensitive to others and remember that things you say may impact on them [42, F6]
Keeping on topic	15	Guidelines that ask members to keep posts relevant, on topic, and provide enough context so others can understand and respond meaningfully.	Keep posts relevant to the group. Please be mindful that this is a mental health group and so to keep your posts mental health related. Nothing inappropriate will be tolerated [62, I3] Discussions in them seldom strayed too far from the designated topic, in part because moderators regularly asked for irrelevant topics to be remove [81, I19]
English language	6	Rules around using the English language and/or the avoidance of slang, abbreviations, or language that may be too complex or exclusive.	All posts must be directly related to mental health, be written in English, well formatted, easily readable and of reasonable length [74, I12] For moderation and inclusive participation, we ask you to please use English in the forum space. Purpose of rule. To create an inclusive and easily accessible space for everyone to participate and understand the discussions that take place in the forums [33, F1]
Boundaries	—	Rules that protect members’ privacy, personal information, and safety, and that define acceptable interactions and contact between members.	—
Sharing personal information	26	Rules prohibiting members from sharing personal information (eg, full names, phone numbers, addresses, email addresses, school or workplace details), and guidelines encouraging members to set boundaries and share thoughtfully to maintain safety.	Do not post content that threatens to reveal personal data or personally identifiable information, including residential address, private email address, private phone number, bank statements, social security numbers and/or passport number [49, F12] In line with Supportive Accountability, users should be encouraged to take responsibility for their own and each other’s safety and consider carefully what information they are posting [10, V5]
Privacy of others	21	Guidelines around members keeping conversations, posts, or interactions private unless explicit permission is given, and rules prohibiting the sharing of another member’s personal or identifying information, whether about a member or someone outside the community, without their consent, as it may compromise their privacy and safety.	Respect each other’s privacy. Do not share outside of the group. Being part of this group requires mutual trust due to some of the sensitive subjects discussed or shared [62, I3] It is not permitted to reveal real names of people or places or to include any information that could lead to individuals being identified with an email [58, F15]
Sexual and romantic communication	15	Rules about sexual or romantic interaction within the community, including flirtation, suggestive language, sexual requests, or attempts to initiate romantic or sexual relationships.	Choose not to engage in sexting or flirting. Graphic, flirtatious, and sexually suggestive language is strictly prohibited [33, F1] Rule 31. Do not post inappropriate/sexual requests or content on TalkLife. This includes sexting requests on the main feed. Replacing characters in the requests (such as ‘s@xting anyone?’) does not make this ok. This also includes sharing explicit details about your sex life or personal fetishes/kinks [49, F12]
Online member contact	12	Guidelines for communicating with other members within the OMHC, including private messaging rules and obtaining explicit permission before sending friend requests or connecting on social media.	Asking to PM/DM (send a direct message) To keep the group safe, and save members from scams, only public conversations are allowed. Do not ask anyone to send a personal message [60, I1] Private Messages (called “Conversations”) are a useful tool for connecting with people in similar situations or even to develop friendships. However, please do not send PMs containing unsolicited advertising, spam, of a sexual nature, or with the intention of entering into a romantic relationship with another member or for the purposes of conducting (formal or informal) online therapy [43, F7]
Offline member contact	9	Rules on meeting offline or exchanging personal details vary across OMHCs, some communities discourage members from meeting in person, while others allow it but strongly advise taking safety precautions if choosing to do so.	Offsite contact is never permitted while you are a member of the 7 Cups community [34, F1] Consider seriously any plans to meet other member/s. It is YOUR responsibility. If meeting, ensure sensible precautions are taken, eg, let someone else know about arrangements, meet in a public place, arrange a limited meeting, ensure that someone meets or contacts you at the end of the meeting [46, F10]
Member wellbeing	—	Guidelines that support members’ safety and mental health.	—
Mindful and responsible sharing of experiences and stories	16	Guidelines that encourage members to share their stories and experiences thoughtfully, focusing on what they’re comfortable disclosing, while recognizing their responsibility for what they choose to share.	Sometimes sharing an intense experience can make us feel exposed and vulnerable, so check in with yourself about why you’re posting, think about what you want to share and how it might make you feel [49, F5] Think before I post. Are you comfortable sharing this information? Remember that you can always delete your own posts if you want to [39, F5]
Taking a break	3	Guidelines encouraging members to step away from the community when they feel overwhelmed or need time for self-care.	The site use rules included. take a break if they feel upset by using the site [22, V10] Know when to take a break: Sometimes it can be helpful to step away from the online community and seek professional support [42, F6]
Usernames	7	Guidelines on what can or cannot be included in usernames, such as avoiding personal identifiers.	Using terms like ’Therapist’ or “Life Coach’ or using Triggering or Inappropriate terms in the username is not allowed [33, F1] Usernames should not contain proper names that, when combined, could be mistaken for someone else [52, F14]
Technology security and safe use	4	Guidance on protecting personal information through secure practices, while also avoiding misuse of apps or any harmful actions like spreading viruses or malicious files.	You must not misuse our Apps by introducing any worms or viruses or any code of a destructive nature [50, F13] We therefore require that you do not. Transfer files that contain viruses, trojans or other harmful programs [52, F14]
Account management	—	Rules governing member accounts.	—
Impersonation	10	Expectations around not pretending to be another person, whether an OMHC moderator, staff member, public figure, professional, or another community member.	Falsifying who you are or creating a second account to harass, attack, deceive or mislead others is not permitted. Claiming to be a site administrator, moderator or staff member is also prohibited. Impersonation will result in an automatic permanent ban [35. F2] 2.5. Do not impersonate other people, either real or fictitious. This includes Wall Guides or other Togetherall staff members. Just be yourself [52, F14]
Multiple accounts	8	Rules requiring each member to use only one account, to ensure transparency and avoid confusion within the OMHC.	Only one member account per individual. People registering more than one account may lose all accounts on the forum, and all forum access [44, F8] Members must only have one account [43, F7]
Sharing accounts	4	Rules around not allowing someone else to use your account or logging into someone else’s account.	Keep your account private - don’t show anyone your password or share your account with someone else [42, F6] It’s not appropriate for several people to share one account, or for accounts to be created on the behalf of someone else [48, F11]
Hunter accounts	3	Rules against creating accounts created solely to monitor, expose, stalk, or provoke other users (sometimes called “hunter” or “catfish” accounts).	Rule 72. Do not take it upon yourself to hunt out predators on the platform [49, F12] Creation of malicious accounts. will not be tolerated [42, F6]
Nonallowed member behavior	—	Rules defining prohibited actions and conduct within the OMHC.	—
Generalization	4	Rules around making broad or harmful statements about groups or individuals, including speaking for others or assuming intentions.	A wide range of people use the Forum, and generalizations may feel excluding [46, F10] Please try to avoid blanket terms/language Using these terms can make people feel excluded and unwelcome, as well as lead to unnecessary harm. Please ensure you’re speaking of your own personal experiences and not on behalf of others [75, I13]
Hate speech	22	Rules around the use of language that attacks, threatens, discriminates against, or dehumanizes someone based on gender, sexuality, disability, religion, race, or culture.	Showing hate towards any gender identity, sexual orientation, disability, religion, racial or cultural background, or imposing your viewpoint on any of these items on another user is forbidden [33, F1] No hate speech. Racist, sexist, homophobic, and other types of hate speech will not be tolerated. Respect everyone for who they are and where they are in life [69, I8]
Offensive language	18	Rules relating to the use of hostile, graphic, or offensive language, such as swearing, vulgar, or crude remarks, and any language that is aggressive, vitriolic, or offensive toward others.	Inappropriate or hostile language will not be tolerated [19, V8] We therefore require that you do not: . Use foul, threatening or offensive language, including, without limitation, racist, sexist, ageist, homophobic or sexually explicit language where inappropriate [52, F14]
AI	2	Rules around using content generated by AI tools.	Personal means no use of AI [70, I9] The excessive use of artificial intelligence when creating forum content is not allowed [33, F1]
Moderation (n=35)^e^			
Reporting posts	36	Guidelines on how members flag or report posts and behaviors that violate community guidelines (n=35), and information on how members reach out to moderators with questions, concerns, or requests regarding the community (n=25), helping moderators maintain a safe and supportive space.	Altitudes includes a ‘report function,’ which enables users at any time to indicate to moderators concerns about any inappropriate material posted by a user [3, V1] The platform also includes safety features such as a reporting function for users [7, V4]
Respecting moderators	12	Expectations within the community for treating moderators with respect, avoiding negative or harmful comments about them, and recognizing their role in maintaining a safe community.	Respect our forum and the people who run it: Please follow these guidelines and the instructions given by the team running the forum [43, F7] We hide posts that talk about moderators individually or as a team [48, F11]

a OMHC: online mental health community.

b Definition: guidelines around any material and content shared or posted by members within the community, including text posts, comments, images, videos, links, or other media.

c Not applicable.

d Definition: guidelines describing how members contribute to the community by creating posts, comments, or replies.

e Definition: guidelines around how members engage with moderators, including asking questions, seeking support, reporting concerns, or responding to moderator guidance.

## Discussion

### Principal Results

This systematized review developed 2 typologies. The typology of identified moderator practices spanned pre- and postmoderation processes, reactive moderation of content, community-building activities, and member-specific monitoring. The community guidelines typology identified 3 categories, comprising the content of individual posts, membership behavior expectations, and moderation approaches.

Both typologies were intentionally designed to be adaptable and flexible in scope. This adaptability is essential for 2 key reasons. First, the typologies were shaped by the content available within the included OMHCs. They therefore reflect the practices and guidelines that were most accessible, rather than offering an exhaustive catalog of all possible moderation approaches. Second, it is essential to acknowledge that the typologies developed through this review are, by necessity, abstractions of complex, real-world phenomena. Rather than prescribing specific actions, the typologies provide examples and categories that can inform thinking, guide discussions, and support the creation of community-specific guidance. Therefore, translating these typologies into practice will require careful adaptation and nuance to the specific goals, purposes, structures, and cultural contexts of individual OMHCs.

### Comparison With Prior Work

The frequency with which certain practices were cited, particularly those related to flagged content, highlights the centrality of safety within OMHCs. This aligns with prior research showing that maintaining safety is a core responsibility of moderators and a cornerstone of community trust and sustainability. For example, a survey conducted across 10 countries involving 13,500 individuals found that 79% of respondents preferred moderating online content for safety rather than prioritizing freedom of speech [98]. Although there were between-country differences, which make a universal approach to moderation difficult, survey findings highlighted the need for moderation practices that are sensitive to the specific values, purposes, and social or cultural norms of each OMHC.

While safety is paramount, the typologies also demonstrate that moderation is a multifaceted process. Moderation encompasses a broad spectrum of responsibilities, extending beyond screening content, signposting to mental health support, fostering community cohesion, guiding user interactions, and shaping a culture. This reflects previous work showing that moderators are not merely gatekeepers but active “shaping forces,” as cultural agents who influence norms, expectations, and culture within their communities [11]. Our findings also highlight how community guidelines operate as both preventive and educational tools. They provide a visible framework that helps members understand what is expected of them and how to participate safely and respectfully. The types of potentially triggering content varied across communities, including where trigger or content warnings were positioned within posts. These findings provide a foundation that other communities can build upon and extend, while recognizing that additional trigger or content warnings exist beyond those identified in this review [99]. Whilst this accounts for expectations surrounding community behavior, consideration of moderator behavior extended beyond the scope of this review. Understanding how moderator behavior (eg, injection of topics, overstepping the moderator role, or taking on a dual role of community member and moderator within an online community) may influence the culture, safety, and sense of community within a group may be an important point of future research.

Differences also emerged regarding language deemed explicit or offensive. Most OMHCs banned such language outright, while others permitted it under specific conditions, such as when it expressed frustration without targeting others. A minority required posts containing explicit language to include trigger warnings. None of the communities published exhaustive lists of prohibited terms, reflecting the inherent fluidity of language and the need for moderator discretion in applying these rules. The availability of community guidelines varied significantly across different platform types. Publicly accessible informal communities, such as those on Reddit (Steve Huffman, Alexis Ohanian, and Aaron Swartz) or Facebook (Meta, Inc), were more likely to publish their guidelines in full. Most intervention-based OMHCs either provided minimal information or did not share their guidelines. While specific guidelines may exist, they may not have been publicly available, possibly to maintain confidentiality or because the details have been published only within academic literature. As a result, the available evidence is skewed towards informal and formal communities, leaving gaps in our understanding of how interventions or closed OMHCs operate. For example, private platforms such as Discord (Discord Inc.) could not be included due to restricted access. This highlights a significant limitation for both research and practice, as some of the most vulnerable populations may be engaging in spaces that are effectively hidden.

Our findings align closely with Skousen et al (2020) [100], who identified 5 categories of practices that members used to moderate successfully in online patient communities: instructive, semantic, connective, administrative, and policing. These categories correspond to our typologies, particularly in areas such as providing support (instructive), facilitating and guiding conversations (semantic and connective), and intervening when rules are breached (policing). Notably, administrative practices focused on community growth were not evident within our analysis, likely because public-facing documents such as codes of conduct do not capture internal strategies or behind-the-scenes activities undertaken by moderators. Although not explicitly stated, the typologies imply that many asynchronous forum moderators take on an instructive role by explaining how to navigate the platform, clarifying its technical features, and signposting users to relevant subforums or resources. For instance, the community guidelines identified issues relating to members holding multiple accounts, though they do not specify how moderators address this. This particular issue around multiple accounts also reflects a broader, increasingly recognized problem in online research [101].

### Limitations

This study has several limitations. First, our search was limited to English-speaking communities and English-language sources, most of which were based in Western countries. This limits the cultural and linguistic reach of the typologies, which may not fully capture the practices of communities operating in other contexts. Adaptation of the findings to other cultures will require adopting, adapting, and releasing inherited norms [102]. Second, because our analysis focused on publicly accessible guidelines, it may not fully reflect the private or intervention-based OMHCs or the behind-the-scenes practices of moderators. For example, intervention-based communities often provided minimal information about moderators’ roles or responsibilities, and closed informal communities were inaccessible to researchers. Third, it did not include substance use–focused communities, which may operate under different moderation policies, user needs, and risk dynamics, thereby limiting the generalizability of the findings across the broader spectrum of online mental health support spaces. Fourth, our search period was 2014 to 2024. An updated MEDLINE search for OMHC publications conducted up to July 16, 2026, identified only 13 further papers, none of which related to moderator practices. Fifth, the absence of formal quality appraisal, combined with purposive gray-literature sampling of community guidelines (using Google and social media platforms only), limits the ability to weight sources and introduces a possible selection effect. Sixth, we used a systematized rather than systematic review methodology. Specifically, protocol registration, exhaustive search strategy, and database searching (eg, using an unprofiled Google search), formal quality appraisal, and analyst reflection were not undertaken. As the goal of this review was to establish the primary features of OMHC approaches to safety and establishing a sense of community, issues of selection and interpretation bias are less relevant, so the extra resources needed for a systematic review were not justified. Finally, while community guidelines provide valuable insight into the rules and expectations governing OMHCs, they may not always map neatly onto moderators’ day-to-day practices. Moderators may engage in nuanced decision-making and relational work that is not explicitly documented in written policies.

### Implications for Practice

The typologies developed in this study provide a foundation for future work to support moderators and strengthen OMHCs. While this review focused on OMHCs, the findings may have broader intersectoral implications.

For developers and moderators, transparent decision-making is essential to ensuring fairness, consistency, and community trust. A key challenge is balancing user safety with the need for open, authentic expression. These sometimes competing goals create ethical and legal dilemmas that require careful judgment. Overly strict or opaque moderation can undermine authenticity, whereas insufficient moderation may expose members to harm. The typologies offer a practical tool and starting point, providing a framework for translating and articulating specific guidelines for moderation decisions, clarifying the moderator role, and supporting practices across different online community platforms. It is noteworthy that only 6 (11.5%) OMHCs had an appeals procedures, suggesting more attention needs to be given to governance arrangements enhancing OMHC trustworthiness. These considerations are especially relevant in the context of evolving social media regulations. Platforms are continually adjusting their policies on freedom of speech, while some countries, such as Australia and the United Kingdom, are introducing restrictions on social media access for children under 16 years. The typologies can help developers anticipate challenges, maintain member safety, and preserve meaningful community engagement in these shifting regulatory contexts. Similarly, the 4 moderator practices identified in Table 1 can provide the basis for a bespoke training package for community members wishing to take on a moderator role, with community-specific guidance for each component, eg, new member screening processes.

In clinical practice, the findings can assist clinicians to recognize safer versus less safe OMHCs. Safer OMHCs are likely to have guidelines for members covering post content and member conduct including many elements from Table 2, and transparent role expectations for moderators spanning premoderation to postmoderation, with member-monitoring and community-building features aligned with Table 1 approaches. This knowledge can inform recommendations for service users seeking online peer support to help them find safe, well-moderated communities. Future research could quantitatively examine whether specific moderation processes are associated with higher engagement, greater satisfaction, or better symptom or psychosocial outcomes. For example, comparing the effects of community-building processes with those centered on member and content monitoring to determine how these different approaches influence user engagement, satisfaction, or outcomes in distinct ways. Research could also examine whether specific moderation processes mediate changes in an individual’s symptoms and psychosocial outcomes. Quantitative studies should be complemented by qualitative approaches such as interviews or ethnographic observation to capture the day-to-day realities, challenges, and decision-making processes involved in moderation.

Finally, our focus on human moderators can inform likely evolution towards automated moderation. Our findings are entirely consistent with a recent independent study which concluded “Moderation is critical in making online peer communities feel safe and engaging” [15] (p.1). Automated moderation of OMHCs is not yet common, but experience from 2 related areas raises concerns. Automated moderation of social media platforms can both reduce some forms of harmful content and create new risks, such as curbing dissemination of harm reduction and health promotion materials through erroneously interpreting it as promotion of drug use and sales [103], or misunderstanding evolving language and failing to recognize “algospeak” used to evade algorithmic moderation [104]. Chatbot therapists have many potential benefits, such as immediate availability and reduced stigma, but also carry risks including addiction, social isolation, misuse of data, hallucinations, bias, and lack of safety [105]. It is known that community moderators develop “knowledge-in-action” which then allows moderator teams to develop “groupwise reflective practice” [106], which becomes self-moderation in a mature community [15]. The contribution, if any, of automated OMHC moderation at each stage will require careful and disinterested evaluation, with equal attention to risks and benefits.

### Conclusions

The typologies presented here suggest that moderation in OMHCs involves a wide range of interrelated responsibilities that extend well beyond the monitoring of content or enforcement of rules. Moderators are required to navigate multiple functions simultaneously, including assessing potential risks to member safety, managing boundary issues, responding to problematic content, facilitating supportive interactions, and fostering community engagement. In some cases, moderators also undertake actions related to safeguarding, such as monitoring individual risk or contacting external services. Taken together, these activities point to moderation as a complex form of sociotechnical and relational work that sits at the intersection of community facilitation, risk management, and mental health support. As many OMHCs rely on peer moderators or individuals with lived experience, this complexity raises important questions regarding the training, supervision, and institutional support required to sustain these roles safely and effectively. Future research should therefore examine how moderators are prepared for these responsibilities and what forms of training, guidance, and organizational structures best support them in balancing community safety with the facilitation of supportive peer interactions. Using ethnographic methods, such as in-depth observation and interviews with moderators in their day-to-day activities, could provide valuable insights into the nuances of moderation processes and the real-world challenges moderators face in carrying out these responsibilities.

As individuals increasingly experience online communities as a central part of their social network, there is a growing need to manage OMHCs well. Our identification of moderator practices and OMHC guidelines contributes to this goal.

## Supplementary material

10.2196/99367 Multimedia Appendix 1 Data extraction form.

10.2196/99367 Multimedia Appendix 2 Extraction Table.

## Abbreviations

EPOCH: Evaluating Positive Changes in Psychosis
OMHC: online mental health community
